# Selenium and/or vitamin E upregulate the antioxidant gene expression and parameters in broilers

**DOI:** 10.1186/s12917-022-03411-4

**Published:** 2022-08-13

**Authors:** Fatma Elgendey, Rasha A. Al Wakeel, Shabaan A. Hemeda, Aya Mohamed Elshwash, Sabreen E. Fadl, Aaser M. Abdelazim, Muhanad Alhujaily, Olla A. Khalifa

**Affiliations:** 1 Genetics and Genetic Engineering, Department of Animal Wealth Development, Faculty of Veterinary Medicine, Benha University, Toukh, 13736 Egypt; 2 Department of Physiology, Faculty of Veterinary Medicine, Kafrelsheikh University, Kafrelsheikh, 33516 Egypt; 3 Department of Animal Husbandry and Animal Wealth Development, Faculty of Veterinary Medicine, Alexandria University, Alexandria, 21526 Egypt; 4 Biochemistry Department, Faculty of Veterinary Medicine, Matrouh University, Matrouh, 51744 Egypt; 5 Department of Basic Medical Sciences, College of Aapplied Medical Sciences, University of Bisha, Bisha, 61922 Saudi Arabia; 6 Department of Medical Laboratory Sciences, College of Applied Medical Sciences, University of Bisha, Bisha, 61922 Saudi Arabia

**Keywords:** Antioxidants, Broilers, Copper and iron, Gene expression, Selenium, Vitamin E

## Abstract

**Background:**

In contrast to free radicals, the first line of protection is assumed to be vitamin E and selenium. The present protocol was designed to assess the roles of vitamin E and/or a selenium-rich diet that affected the blood iron and copper concentrations, liver tissue antioxidant and lipid peroxidation, and gene expression linked to antioxidants in the liver tissue of broilers. The young birds were classified according to the dietary supplement into four groups; control, vitamin E (100 mg Vitamin/kg diet), selenium (0.3 mg sodium selenite/kg diet), and vitamin E pulse selenium (100 mg vitamin/kg diet with 0.3 mg sodium selenite/kg diet) group.

**Results:**

The results of this experiment suggested that the addition of vitamin E with selenium in the broiler diet significantly increased (*P* ≤ 0.05) serum iron when compared with the other groups and serum copper when compared with the vitamin E group. Moreover, the supplements (vitamin E or vitamin E with selenium) positively affected the enzymatic activity of the antioxidant-related enzymes with decreased malondialdehyde (MDA),which represents lipid peroxidation in broiler liver tissue. Moreover, the two supplements significantly upregulated genes expression related to antioxidants.

**Conclusion:**

Therefore, vitamin E and/or selenium can not only act as exogenous antioxidants to prevent oxidative damage by scavenging free radicals and superoxide, but also act as gene regulators, regulating the expression of endogenous antioxidant enzymes.

## Background

A combination of stressing factors (environmental, nutritional, technological, and individual) has been blamed for decreased poultry welfare [1], production performance, and bird immune response [2, 3]. Fertility and hatchability rates are affected by stress conditions [1]. Moreover, growing chicks display poor feed conversion, decreased average daily weight growth, immunosuppression, and higher mortality if experiencing stress. A number of investigations have pointed to the effects of stress at the cellular level as a result of excessive free radical generation or insufficient antioxidant defense [4]. The excessive buildup of free radical species (ROS/RNS) is followed by disruption in cell homeostasis, which leads to oxidative damage such as lipid peroxidation and oxidative damage to proteins and DNA [5–8]. Antioxidant defense systems are made up of a complicated network of antioxidants that are synthesized internally as enzymes and supplied externally as vitamins and minerals [7]. However, the cell has three levels of protection against these free radicals [9–11], the first one is represented by enzymes that have antioxidant activity such as catalase (CAT), glutathione peroxidase (GPx), and superoxide dismutase (SOD). These enzymes are detoxify free radicals at an early stage of formation [12]. Furthermore, metal-binding proteins are considered in the first level of antioxidant defense, where free iron and copper are the most important catalyzers of free radical formation. The second level includes vitamin E, glutathione (GSH), and ascorbic acid, which are known as free radical scavenging antioxidants. However, of the fat-soluble vitamins, it is vitamin E that is considered the major cell membrane antioxidant. The third level deals with the damaged molecule’s repair as enzymes that repair DNA or removal as phospholipases and proteasomes. Thus, internal antioxidant systems reflect the adaptation/coping ability of birds, but when endogenous systems are not enough, stress leads to poor welfare and health [7]. As a result, for poultry scientists, developing efficient nutritional treatments to reduce the deleterious consequences of commercially relevant stressors is a critical challenge. Thus, in numerous biochemical and physiological processes, including antioxidant activity, tocopherols serve a critical role [13, 14]. Moreover, Tawfeek et al. [15] found vitamin E in the broiler diet improves the response of immunity and reduces chicken mortality when infected experimentally with *E. coli*. *Apostichopus japonicus’s* growth and antioxidant defences may respond favorably to Se and vitamin E supplementation [16]. Selenium is an important micro mineral essential for the growth of poultry [10, 11, 13–15, 17]. Zhang et al. [18] reported the antioxidant effect of selenium supplementation. Muhammad et al. [19] studied the effects of different sources of Se on hepatic total antioxidant capacity, GSH-Px, and CAT activity, which are all increased by selenium administration. The addition of selenium in animal feeds increases the animal’s immune state and the ability of the immune system to respond to experimental challenges [20]. Moreover, selenium is required for GPx, which transforms H_2_O_2_ and lipid hydroperoxides into alcohols [21, 22]. Vitamin E and selenium, in particular their antioxidant and immune functions, can work together to influence biological processes [23, 24].

In contrast to free radicals, the first line of protection is assumed to be vitamin E and selenium, although the synergy between them is still unclear. Therefore, this experiment was done to discover the roles of vitamin E and selenium supplementation individually or together on the liver antioxidant enzymes, some elements in the serum, and antioxidant-related gene expression in broilers’ liver tissue.

## Results

### Serum iron and copper

Vitamin E or selenium supplementation in the broiler’s diet affects iron and Cu concentrations (Table 1). Statistical analysis of the obtained data revealed that Fe concentration significantly increased (*P* ≤ 0.05) when vitamin E with a selenium-enriched diet was used in comparison with the other groups. Meanwhile, a vitamin E enriched diet insignificantly increased (*P* ≤ 0.05) serum Fe concentration, while a selenium-enriched diet insignificantly (*P* ≤ 0.05) reduced the same parameter in comparison with the basic diet group. On the other hand, statistical analysis of the collected data revealed that including vitamin E in the diet significantly reduced (*P* ≤ 0.05) serum Cu concentration when compared with the control and vitamin E with selenium groups.

**Table 1:** Effect of vitamin E and/or selenium-enriched diet on broiler serum iron and copper concentrations

Groups Items	Control	Vitamin E	Selenium	Vitamin E with selenium
Fe (μg/L)	64.80±3.38^b^	69.63±1.56^b^	61.30±2.12^b^	84.93±3.24^a^
Cu (μg/L)	91.97±1.25^a^	83.60±3.41^b^	90.13±1.92^ab^	94.23±1.94^a^

Values are means ± standard error. Mean valueswith different letters (a-b) at the same row significantly at (*P* ≤ 0.05)

### Liver tissue antioxidant and MDA

Liver antioxidant enzyme activities and MDA concentrations in broiler chickens are shown to be affected by dietary vitamin E or selenium supplementation (Table 2). When the collected data was statistically analyzed, it was discovered that a vitamin E-rich diet and vitamin E combined with a selenium-enriched diet significantly (*P* ≤ 0.05) increased the activities of the liver tissue CAT and SOD when compared to the control group. Meanwhile, the supplementation of selenium insignificantly increased (*P* ≤ 0.05) the activities of the liver tissue CAT and SOD when compared with the group with a basic diet. On the other hand, statistical analysis of the gained data showed vitamin E inclusion alone or with selenium in the diet significantly reduced (*P* ≤ 0.05) the MDA concentration in the tissue of the liver in comparison with the group with no supplement. Moreover, the supplementation of selenium statistically insignificantly decreased (*P* ≤ 0.05) liver tissue MDA concentration when compared with the group with a basic diet.

**Table 2:** Effect of vitamin E and/or selenium-enriched diet on broiler liver tissue antioxidant and MDA

Groups Items	Control	Vitamin E	Selenium	Vitamin E with selenium
CAT (U/g)	0.73±0.02^c^	0.87±0.01^b^	0.76±0.01^bc^	1.28±0.08^a^
SOD (U/g)	46.15±0.84^b^	58.70±2.35^a^	46.23±2.40^b^	61.67±0.75^a^
MDA (nmol/g)	3.69±0.32^a^	1.96±0.17^b^	3.67±0.37^a^	1.25±0.00^b^

Values are means ± standard error. Mean values with different letters (a-c) at the same row significantly at (*P* ≤ 0.05)

### Expression levels of antioxidant enzyme genes

**Fig. 1:**
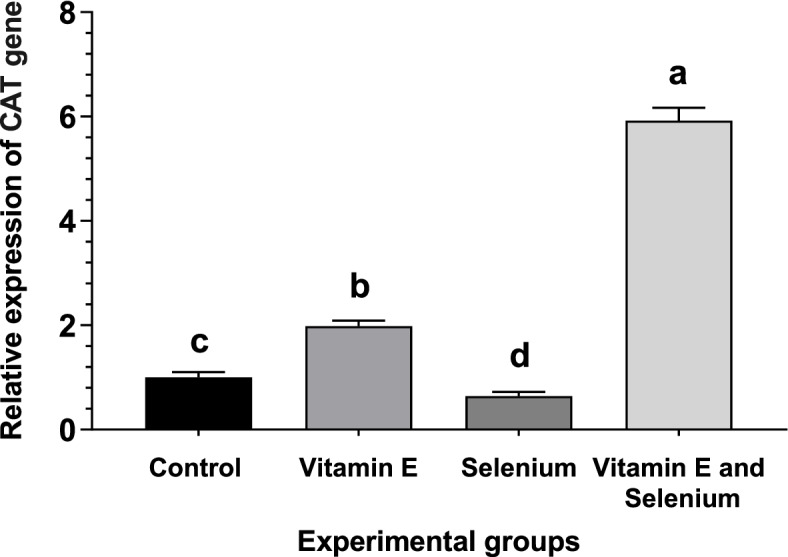
Effect of vitamin E and/or selenium-enriched diet on broiler liver *CAT* mRNA transcript level

**Fig. 2:**
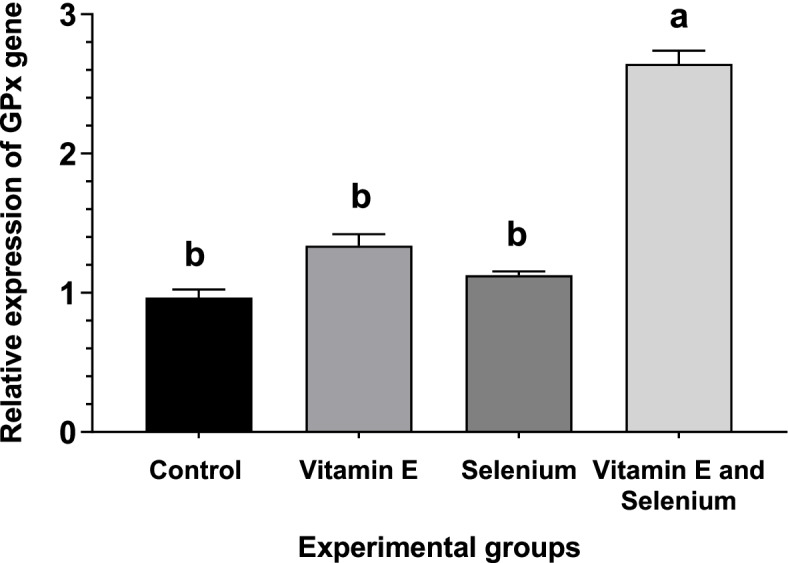
Effect of vitamin E and/or selenium-enriched diet on broiler liver GPx mRNA transcript level

**Fig. 3:**
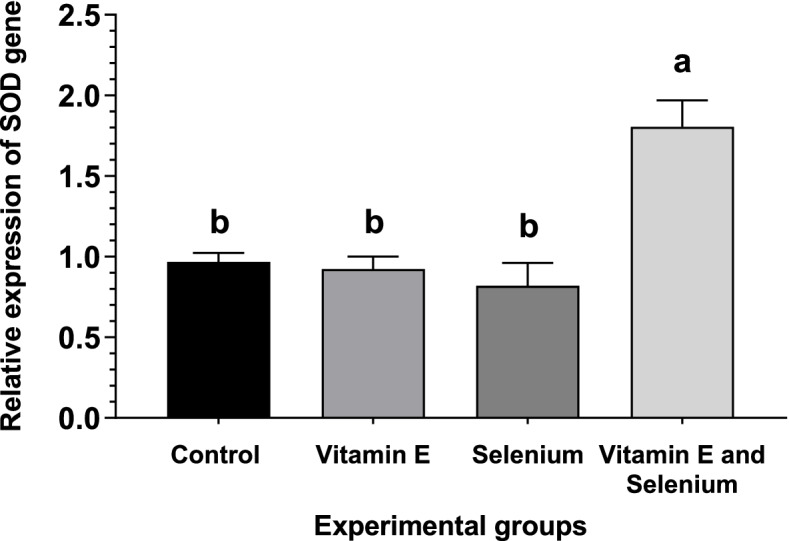
Effect of vitamin E and/or selenium-enriched diet on broiler liver SOD mRNA transcript level

The expression of antioxidant enzymes as affected by vitamin E supplementation with or without selenium in broilers are shown in Figs. 1, 2, and 3. There was a change in the expression pattern of *CAT*, glutathione peroxide (*GPx*), and *SOD* after the addition of selenium and vitamin E to the diet. The supplementation of vitamin E with selenium-enriched diets resulted in a substantial increase in antioxidant enzyme levels, as evidenced by the upregulation of *CAT, SOD*, and *GPx* genes. Moreover, the vitamin E enriched feed upregulated *CAT* and *GPx* genes in comparison with the basic and selenium-enriched diet.

## Discussion

Animal bodies have evolved complex systems to cope with an excess of free radicals created by oxidative stress to preserve redox equilibrium [25]. These defense systems either scavenge or detoxify ROS, prevent their formation, or sequester free radical-producing transition metals. It includes endogenous and exogenous antioxidants where endo is produced in the body and includes enzymatic and non-enzymatic [26] while Exo is dietary [27]. Non-enzymatic antioxidants, such as glutathione and vitamins E, A, and C are crucial in scavenging ROS [28–30]. Meanwhile, enzymatic antioxidants convert free radicals to less damaging compounds [31]. The most well-known enzymatic antioxidants are SOD, CAT, and GSH-Px, which are considered the first-line defense against ROS [32]. The results of the serum Fe and Cu concentrations in the present investigation are confirmed by the results of Sahin et al. [33, 34], who reported that dietary inclusion of vitamin E increases and decreases the concentrations of Fe and Cu, respectively, in the serum of broilers. Furthermore, Harsini et al. [35] observed the same results with the vitamin E and selenium-enriched diet in broilers under heat stress, but there is no effect for this enriched diet at normal temperature. These results may be attributed to the dietary supplementation of vitamin E. As a result of dietary vitamin E, iron is released into the serum, which raises the serum iron content [33, 36]. On the other hand, Kutsky [37] and Van Saun [38] reported the opposite relationship between vitamin E and Cu concentration. Moreover, antioxidants from vitamin E and a selenium-enriched diet may have contributed to these outcomes. Ions of copper are considered potent catalysts for free radical damage. These copper ions can cause oxidative damage through the Haber-Weiss reaction, due to the formation of a highly reactive hydroxyl radical (OH ^−^) [39–41]. The liberated OH^−^ leads to lipid peroxidation, which is represented by MDA [41, 42]. Thus, in this investigation, it was noticed that supplementation of vitamin E significantly decreased serum copper. This point needs more investigation to know the relationship between serum copper concentration and vitamin E supplementation. Vitamin E supplementation, on the other hand, increased serum Fe concentration, which is the main component of haemoglobin in RBCs [43, 44]. It is needed for the transportation of oxygen all over the body through hemoglobin and myoglobin [45, 46] for the delivery, storage, and use of oxygen in muscles [47]. Both hemoglobin and myoglobin are required for normal meat color that is needed as an indicator for meat quality [48]. These results were confirmed by the results of the antioxidant enzymes and lipid peroxidation represented in MDA.

The process of free radical formation and antioxidant is complicated where oxygen is a critical component of animal life, but when it exceeds become poisonous. Additionally, free radicals are produced normally and continuously under normal physiological conditions, but in stress conditions, their production increases in the cell. Free radicals are produced mainly through the mitochondrial electron transport chain, xenobiotic-metabolizing enzymes, and immune cells, especially immune cells that generate free radicals to kill the pathogen. On the other side, developing an antioxidant system in an oxygenated environment is an adaptive evolutionary process for survival. The data of the liver antioxidant enzymes are in harmony with the results of Karadas et al. [13], who reported that dietary addition of selenium and vitamin E increases the accumulation of antioxidants and decreases MDA in the liver tissue of broilers. These results are attributed to Vit E supplementation, which is known as an antioxidant of the membrane that lessens the negative effects of free radicals and reactive oxygen species that would otherwise lead to the oxidation of crucial sulphydryl groups and phospholipids [49]. Thus, MDA concentration was decreased and antioxidants were increased in our study. Moreover, Coskun et al. [50] and Xu et al. [16] found that selenium and vitamin E have beneficial effects on the antioxidant responses of *Galleria mellonella L.* and *Apostichopus japonicas,* respectively. Moreover, Gouda et al. [51] reported that supplemental vitamin E and/or selenium improve antioxidant enzyme activity with decreased lipid peroxidation. Melčová et al. [52] reported that vitamin E supplementation in rats increased liver CAT. Broilers fed diets deficient in vitamin E and selenium display the lowest levels of reduced glutathione and glutathione peroxidase activity [53]. The same results were noticed by Sahin et al. [33] dietary vitamin E decreases serum and liver MDA in heat-stressed broilers. These results may be attributed to the ability of vitamin E to neutralize free radicals and reduce lipid peroxidation [54]. Surai [55] reported that vitamin E (Toc-OH) effectively scavenges peroxyl radicals (ROO*) and done (Toc-O*) and (ROOH). Meanwhile, Se is required for the activity of GSH-Px, which needs adequate Se in the cell even in the presence of very high levels of vitamin E in the diet [56, 57]. As a consequence of antioxidant gene expression in liver tissue, these results were verified.

The expression of antioxidant enzymes in the liver tissue is confirmed by the results of Kumbhar et al. [58], who found that dietary supplementation of selenium and/or vitamin E enhances the mRNA of *CAT* and *SOD* levels. The expression levels of *CAT, GPx, and SOD* are elevated in the liver tissue of broilers with increasing selenium levels in their diet [59]. Niu et al. [60] found that *SOD* and *GSH-Px* mRNA expression are increased with dietary vitamin E supplementation, which consequently increases the quality of meat through upregulation and expression of antioxidant genes in broilers. Moreover, vitamin E administration to hypothyroid rats leads to an elevation of *CAT* mRNA levels [61]. Moreover, Cu/Zn *SOD* and *CAT* mRNA levels increase in human umbilical vein endothelial cells with Vit E [62]. These results indicate that vitamin E can not only play a role in preventing oxidative damage through scavenging free radicals and superoxide as an exogenous antioxidant but can also modulate the expression of endogenously produced antioxidant enzymes as gene regulators. The findings of gene expression were confirmed by the results of the antioxidant enzymes and lipid peroxidation represented by MDA in broilers’ liver tissue.

## Conclusion

The findings of this research indicated that the vitamin E and selenium-enriched diets individually or together significantly (*P* ≤ 0.05) upregulated the expression of the antioxidant genes. Thus, a diet enriched with vitamin E and/or selenium can not only play a role in preventing oxidative damage through scavenging free radicals and superoxide as an exogenous antioxidant but can also modulate the expression of endogenously produced antioxidant enzymes as gene regulators. Moreover, the supplements positively affected the activities of the antioxidant enzymes with decreased lipid peroxidation in the liver tissue of broilers.

## Materials and methods

### Ethical approval

Egypt’s Benha University’s Faculty of Veterinary Medicine has approved the present work (BUFVTM 04–06-21).

### Chemicals

Vitamin E was obtained in the form of alpha-tocopherol acetate (It is a semisynthetic acetate ester of naturally occurring α-tocopherol) from Sigma Aldrich Co., USA. Selenium was obtained in the form of sodium selenite from Eibico Company, Egypt. Sigma Aldrich Co., USA, provided the N-ethylmaleimide. Biodiagnostics Company provided all biochemical analysis kits (Dokki, Giza, Egypt).

### Birds and design of the experimental

Ninety-six (unsexed one-day-olds with an average body weight = 51.72 ± 0.17 g/chick) broilers (Cobb-505) were taken from a farm located in Benha city, Egypt. We were informed that consent was obtained from the owners for handling the animals. The chicks were housed in a well-ventilated room with free access to food and water. The chicks were raised in a clean and sanitary environment. The experimental birds were randomly distributed into 4 groups by the ranking method, with three replicates/group (8 birds/replicate). A designed basal diet was provided to the control group, which was prepared in accordance with Broiler Nutrition Specification, 2007 (Table 3). The vitamin E group fed on the formulated diet contains vitamin E (100 mg -kg diet). The selenium group fed on the formulated diet contains inorganic selenium (0.3 mg -kg diet). The vitamin E and selenium group fed on the formulated diet contains both vitamin E and inorganic selenium. The birds were reared for 30 days, and all vaccination programmes were done during this period. All procedures were followed in compliance with relevant guidelines and regulations. Moreover, the authors affirm that the research was conducted in accordance with the ARRIVE guidelines.

**Table 3:** Ingredients and calculated chemical composition of the used experimental diets

Physical composition	Basal diet (0–3) weeks	Basal diet (3–5) weeks
Yellow corn	57.13	63.86
Soybean meal 48%	29	25
Corn glutine 62%	6.5	4
Sunflower oil	3.2	3.5
Dicalcium phosphate	1.85	1.35
Lime stone	1.25	1.32
Choline 60%	.22	.17
Lysine	0.03	.06
Methionine	0.12	.04
Common salt	0.4	0.4
Premix^a^	0.3	0.3
**Chemical composition %**		
ME Kcal/kg	3177.85	3234
Crude protein	23.066	20
Calcium	1	.9
Available phosphorus	0.45	0.35
Lysine	1.1	1
Methionine + cystine	0.9	.72
Cholin	1300 mg/1 kg	1000 mg/1 kg

^b^The used premix provides Vit A (12,000 Iu), vit D (5000 Iu), vit E (50 mg), vit K3 (3 mg), vit B1 (3 mg), vit B2 (8 mg), vit B6 (4 mg), vit B12 (0.016 mg), nicotinic acid (60 mg), pantothinic acid (15 mg), folic acid (2 mg), biotin (0.2 mg), iron (40 mg), copper (16 mg), zinc (100 mg), manganese (120 mg), iodine (1.25 mg), selenium (0.3 mg) per 1 kg diet

### Samples collection

After 30 days, the blood samples were collected (left for 20 min for coagulation before separation) from the wing vein without anticoagulant (5 broilers/group) after anesthesia. The anaesthesia was done by following the Facility Animal Care Committee of Veterinary Medicine - Benha University, Egypt, that approved protocols and institutional policies. Each bird was injected separately and handled softly and carefully to reduce stress for both the animal and the operator. Where exactly 50 mg/kg of sodium pentobarbital was used intraperitoneal (IP) for the induction of anesthesia. The birds were taken randomly. At 4 °C, serum samples were separated by spinning of blood samples at 3000 rpm for 15 minutes and then refrigerated at − 20 °C to utilize in analysis. The serum had been diluted with deionized water for measuring copper (Cu) and iron (Fe) concentrations using an atomic absorption spectrophotometer (Perkin–Elmer, AA- 600, USA) according to Helrich [63]. The liver tissue samples (1 g/sample) were collected by using an overdose of anesthesia and kept at − 80 °C until used.

Using an electrical homogenizer surrounded by ice, cold phosphate buffer (10 ml) pH 7.4 was used to homogenize liver tissue samples. The homogenized tissues were centrifuged at a speed of 5000 rpm for 30 min [64]. According to Nishikimi et al. [65], Aebi [66], and Uchiyama and Mihara [67], the supernatant was utilized to measure the activities of SOD and CAT, as well as the levels of MDA, respectively.

#### RNA isolation and reverse transcription

The birds (*n* = 5) from each group were chosen by a random method. Liver specimens of 30 mg from each bird were used for suspending in RNA lysis buffer, which contains β-mercaptoethanol and were homogenized. This step was done using a Tissue Lyser that was obtained from Qiagen, Hilden, Germany. Total RNA was collected using the RNeasy mini kit (QIAGEN) according to the manufacturer’s instructions after a 2-minute high-speed (30 Hz) shaking step. Nano drop ND1000 (Uv-Vis spectrophotometer Q5000/USA) was used to estimate the concentration of total RNA at 260/280 nm and kept at − 80 °C.

Table 4 shows the gene-specific primer Metabion obtained from Martinsried, Germany, that was used in this trial. Reverse-transcription from RNA to cDNA was done. PCR Master Mix was carried out according to the Quanti Test SYBR green PCR kit (QIAGEN). The 25 μl reaction mixture consisted of 12.5 μl of 2x QuantiTect SYBR Green PCR Master Mix reaction, 0.5 μl of each forward and reverse primer (20 pmol), 7 μl of RNA, 0.25 μl of reverse transcriptase, and 4.25 μl of RNAase-free water. The thermal cycling conditions of qRT-PCR for primer annealing and subsequent melting curve analysis were: reverse transcription at 50 °C for 30 min, initial denaturation at 94 °C for 5 min, followed by 40 cycles, denaturation at 94 °C for 15 s and annealing for 30 s at 60 °C. Finally, the 30s of extension at 72 °C was done.

**Table 4:** Primers used for qRT-PCR analysis (*n* = 5)

Gene	Primer	Ref.	Annealing
*β-actin*	F: ACCTGAGCGCAAGTACTCTGTCT R: CATCGTACTCCTGCTTGCTGAT	[68]	60
*SOD*	F:CGGGCCAGTAAAGGTTACTGGAA R:TGTTGTCTCCAAATTCATGCACATG	[69]	60
*CAT*	F: ACTGGTGCTGGCAACCC R: ACGTGGCCCAACTGTCAT	[69]	60
*GPX*	F: GCGACTTCCTGCAGCTCAACGA R: CGTTCTCCTGGTGCCCGAAT	[70]	60

*SOD* superoxide dismutase, *CAT* catalase, *GPx* glutathione peroxidase

Analysis of the dissociation curve was done by one thermal cycle of secondary denaturation at 94 °C for 1 min, annealing at 60 °C for 1 min, and final denaturation at 60 °C for 1 min. The genes were checked in duplicates for the five birds. The mRNA quantity was calculated in relation to the expression of the β-Actin reference gene. The SYBR green quantitative real-time polymerase chain reaction (qRT-PCR) was analyzed through amplification curves. Then Ct values were calculated by the Stratagene MX3005P software. To estimate the change of mRNA expression for different samples, the Ct of each sample was compared with that of the control group following 2^−ΔΔCT [71].

### Statistical analysis

The result of each finding was given as mean ± standard error. Version 20 of SPSS was used to analyze the data. Where we used one-way ANOVA followed by Duncan’s post hoc test for multiple groups comparison to see how vitamin E and/or a selenium-rich diet upregulate the antioxidant gene expression and parameters of broilers. The significance value was *P* ≤ 0.05.

## Data Availability

The datasets supporting the conclusions of this article are included within the article.

## Abbreviations

CAT: Catalase
DNA: Deoxyribonucleic acid
GPx: Glutathione peroxidase
GSH: Reduced glutathione
Q-PCR: Real-Time Polymerase Chain Reaction
MDA: Malondialdehyde
RNA: Ribonucleic acid
Se: Selenium
SOD: Superoxide dismutase
